# 718 Indirect Calorimetry is Necessary to Optimize Nutrition in Large Burns

**DOI:** 10.1093/jbcr/irac012.272

**Published:** 2022-03-23

**Authors:** Shruthi Srinivas, Sheela S Thomas, Alfredo C Cordova, Nicole O Bernal

**Affiliations:** Ohio State University, Columbus, Ohio; The Ohio State University Wexner Medical Center, Dublin, Ohio; The Ohio State University Wexner Medical Center, Dublin, Ohio; The Ohio State University Wexner Medical Center, Columbus, Ohio

## Abstract

**Introduction:**

Assessing nutritional requirements in large total body surface area (TBSA) burns is a challenge due to frequent metabolic variation. Studies have compared indirect calorimetry (IC) with predictive equations and identified formulas that are frequently used in the absence of IC. Large TBSA burns remain poorly understood, as does the role of changing metabolic rate and multiple surgical procedures over the course of recovery. We sought to understand whether these equations remain reliable in these situations.

**Methods:**

The patient is a 31 year old, 92% full-thickness TBSA burn who was studied over the first 75 days of hospitalization at various points per institutional IC guidelines. Using total energy expenditure (TEE) by IC as the standard, we assessed variation of each predictive equation for accuracy. Only the Milner and Toronto formulas are dynamic, and we analyzed variation by post-burn day (PBD) compared to dates of major surgical procedures in these studies. The patient was excised on PBD 1, 2, 5, 7, 9, 21, and 25; he was grafted on POD 5, 11, 20, 41, and 56. Weekly monitoring of prealbumin and CRP indicated adequate nutrition.

**Results:**

On post-burn day (PBD) 5, when all major burns were excised, all predictive equations inappropriately estimated the patient’s energy expenditure. On longitudinal analysis of 16 IC studies (**Table 2**), the Milner equation was most accurate, estimating within 5% variance of TEE at 5 time points (31%). The Toronto formula did not estimate within 5% variance at any time point. No energy equation consistently and accurately estimated energy expenditure over all calculated time points.

**Conclusions:**

Although predictive equations are frequently used, in high TBSA burns with many operations and changing nutritional needs, equations are not as accurate as IC. Given that over- or under-estimating needs result in many complications avoided with IC, we propose frequent IC for intubated, high TBSA patients.

